# Excited Catatonia — A Delayed Neuropsychiatric Complication of COVID-19 Infection

**DOI:** 10.7759/cureus.13891

**Published:** 2021-03-15

**Authors:** Sultan M Zain, Purushothaman Muthukanagaraj, Nishath Rahman

**Affiliations:** 1 Department of Psychiatry and Internal Medicine, United Health Services, Binghamton, USA; 2 Department of Psychiatry, Burrell College of Osteopathic Medicine, Las Cruces, USA

**Keywords:** catatonia, covid-19 neuropsychiatric manifestations, covid-19, post-covid sequelae, first episode psychosis

## Abstract

An increasing number of patients have been presenting with neuropsychiatric signs and symptoms associated with coronavirus disease (COVID-19). We present a case of a 69-year-old female with no prior psychiatric history who was brought to the emergency department due to bizarre behavior and paranoid thoughts for four to six weeks, worsening over the last two weeks. Psychiatric evaluation found that the patient had extreme restlessness and agitation, poor eye contact, paranoid delusions, visual hallucinations, and a flat affect with stereotypic repetition of speech and loose associations. The patient’s family noted that two months prior she had symptoms of common cold associated with a severe cough and 20 pounds of weight loss. Suspicion for prior COVID-19 infection prompted an IgG antibody test, which was positive. Our patient displayed at least three of the signs needed to diagnose catatonia - agitation, rigidity, and echolalia - and had a therapeutic response to lorazepam, confirming suspicions of excited catatonia. Her seropositivity for IgG against COVID-19 suggested a COVID-induced brief psychotic disorder with catatonia, which makes this the first known case, to our knowledge, of a patient with delayed onset catatonia after COVID-19 infection. This suggests that clinicians should, after ruling out more plausible stressors, suspect possible coronavirus involvement in sudden onset psychotic disorders, especially in patients who do not fit the demographic of new-onset schizophrenia-spectrum diagnoses. Further research is needed on the pathophysiology behind COVID-19 altering neuronal function and neurotransmitter pathways.

## Introduction

Given its varied clinical presentation, often involving far-reaching effects on multiple body systems, coronavirus disease (COVID-19) has presented the medical community with an array of diagnostic and therapeutic challenges. As the pandemic continues, we have seen an increasing number of patients presenting with neuropsychiatric signs and symptoms [1,2]. We present the first known case of delayed, new-onset psychosis with excited catatonia after COVID-19 infection in a patient with no prior psychiatric history.

Catatonia is primarily characterized as a dysregulation of motor function but is associated with a heterogeneous presentation involving a wide variety of alterations in mood, behavior, and thought process/content. The *Diagnostic and Statistical Manual of Mental Disorders, Fifth Edition* (DSM-5) indicates that at least three of the following should be present for a diagnosis of catatonia to be made: catalepsy, waxy flexibility, stupor, agitation (in the absence of external stimuli), mutism, negativism, posturing, mannerisms, stereotypies, grimacing, echolalia, and echopraxia [3]. Catatonia can not only be attributed to or occur in conjunction with psychiatric conditions such as depression and schizophrenia but also occur secondary to general medical conditions or toxic syndromes [4]. There are subtypes of catatonia, differentiated primarily by the nature of dysregulation in motor function; the most common type is retarded catatonia followed by excited catatonia and malignant catatonia. Excited catatonia, as exhibited in this case, is accompanied by severe psychomotor agitation and restlessness, pressured speech, and/or combativeness [4,5]. The lorazepam test, involving administration of IV lorazepam at 1 or 2 mg, temporarily relieves symptoms of catatonia, as seen in this case, and is considered as verification of the diagnosis [3]. Benzodiazepines are the first-line therapy for catatonia; electroconvulsive therapy (ECT) and N-methyl-D-aspartate (NMDA) antagonists like amantadine can also be used in refractory cases [6]. While atypical antipsychotics can be used, due to the risk of neuroleptic malignant syndrome (NMS) they pose in catatonic patients, their use is not recommended [6]. 

## Case presentation

A 69-year-old Caucasian female with no prior psychiatric history was brought to the emergency department for displaying bizarre behavior, confusion, and paranoid thoughts for four to six weeks, worsening over the past two weeks. She was paranoid that the government was tracking her and that her husband had drugged her and bugged her phone. The patient’s husband and son indicated that the patient began displaying erratic behaviors four to six weeks ago that had gotten worse in recent days. Her agitation and paranoia were stated to be more pronounced at night. She had not been sleeping well in the prior three days, sleeping an average of one to two hours per night, compared to her normal nine-hour sleep routine. The husband denied that the patient uses any recreational drugs or alcohol. The patient's past medical history was significant for chronic obstructive pulmonary disease, type 2 diabetes, and hypertension. 

Upon presentation, Glasgow Coma Scale was 15 and vitals showed a blood pressure of 174/87, pulse of 85, respiratory rate of 14, temperature of 37.7 °C, and oxygen saturation of 100% on room air. Laboratory investigations revealed a blood urea nitrogen (BUN) of 29 mg/dL, creatinine of 0.4 mg/dL, aspartate aminotransferase (AST) of 68 U/L, and alanine aminotransferase (ALT) of 38 U/L. Serum ethanol level and urine drug screen were negative. Complete blood count revealed no abnormalities. Creatine kinase (628 U/L) was found to be elevated. Polymerase chain reaction (PCR) tests for COVID-19 and influenza were negative. Chest X-ray and computed tomography (CT) scan of the head without contrast revealed no abnormalities. Urinalysis was normal. Troponin I was mildly elevated at 0.147 ng/mL but the patient denied any chest pain, shortness of breath, diaphoresis, or palpitations. Electrocardiogram (EKG) demonstrated nonspecific ST-segment abnormalities. Due to the elevated troponin, the patient was admitted to the medical unit and a non-ST elevation myocardial infarction (NSTEMI) workup was done. Troponins were trended (0.147 ng/mL, 0.163 ng/mL, 0.129 ng/mL) and echocardiogram revealed no abnormalities. In light of these findings, the possibility of this being a cardiac event was deemed low. 

Later on, the patient’s family reported that two months prior, both the patient and her husband had suffered symptoms of the common cold associated with a severe cough and twenty pounds of weight loss. Age-appropriate preventive cancer screening was up to date. The patient had not gotten tested for COVID-19 then and her polymerase chain reaction (PCR) test for COVID-19 at the time of admission was negative. Hence, an antibody test was done on day six of admission, which came back positive for IgG antibodies against COVID-19. Her initial complete blood count (CBC) had shown a normal white blood cell (WBC) count (6.1k/uL) and C-reactive protein was also normal (<0.9 mg/dL), indicating that an acute inflammatory state was unlikely.

During the first few days of hospitalization, though the patient was alert and oriented, she displayed extreme agitation and aggressive behavior, intermittently necessitating restraints. On day three, psychiatry was consulted due to the patient's severe paranoia. It was noted that she had some rigidity on physical exam, was extremely restless and agitated, demonstrated poor eye contact, and had a flat affect with echolalia and loose associations. 

While delirium and dementia were considered, the patient was alert and oriented to time, place, and person, making these diagnoses less likely. Furthermore, her symptoms had been present for weeks, which did not rule out delirium but made it less likely. Due to the patient’s agitation, rigidity, and echolalia, psychiatry noted their concern for excited catatonia and suggested a lorazepam challenge test with 1 mg of lorazepam, which led to an improvement in symptoms. As such, it was suggested that lorazepam 0.5 mg three times daily should be started. 

The patient’s agitation and paranoia appeared to improve with the administration of benzodiazepines, supporting a diagnosis of acute psychosis with excited catatonia. Later, haloperidol 2 mg daily, which had been started by the medicine service, was tapered off and stopped as it was overly sedating the patient. Furthermore, given that typical antipsychotics like haloperidol have been found to worsen catatonia and/or increase the risk of NMS, it was decided that monotherapy with a benzodiazepine would be preferred. Lorazepam 0.5 mg three times daily was switched to clonazepam 1 mg twice daily for dosing convenience. Psychiatry reevaluated the patient on day 10 and noted that, while the patient had improved with respect to symptoms of anxiety, agitation, delusions, and paranoia, she continued to display delusions and appeared to be having visual hallucinations. The patient continued to show gradual improvement and was transferred from the hospitalist service to psychiatry on day 13. By day 14, while she was still mildly anxious and paranoid, she was no longer having paranoia regarding her husband and was showing better insight regarding her condition. Following this, she was discharged with clonazepam 0.5 mg orally twice daily as needed for anxiety. On follow-up, her symptoms were noted to have completely resolved and she was found to have regained most of her lost weight.

## Discussion

Neuropsychiatric symptoms have been noted even in prior outbreaks of coronavirus such as those of severe acute respiratory syndrome (SARS) and Middle East respiratory syndrome (MERS) [1]. Not surprisingly, given the neurotropic characteristics COVID-19 shares with the aforementioned strains, it has been found to cause similar symptoms, especially with respect to delirium, which in some patients can be the only clinical manifestation of COVID-19 [1,2]. While delirium may be a fairly common complication or presenting feature of COVID-19, instances of catatonia associated with COVID-19, while relatively rare, have also been noted. 

There have been a number of cases documented in the literature of catatonia occurring in association with COVID-19 infection [7-12]. One case documented retarded catatonia with concurrent COVID-19 infection in a patient with no prior significant history; the authors attributed the presentation to possible brief psychotic disorder in the setting of increased anxiety secondary to the pandemic, involvement of medications associated with catatonia, as well as the direct neurologic impact of infection [7]. In a similar case of a patient with no prior psychiatric history, catatonia was diagnosed co-occurring with delirium [8]. COVID-19 has also been found to exacerbate psychosis and potentially cause catatonia in patients with a prior psychiatric history. In one case, a patient with schizophrenia had an increase in pro-inflammatory markers leading the authors to note that an inflammatory state could be associated with altered GABAergic transmission and a hypodopaminergic state in the basal ganglia [9]. Another patient with a history of schizophrenia presented with a sudden exacerbation of psychotic symptoms and onset of catatonia in the setting of COVID-19 infection; since the patient was also hyponatremic, the authors postulated that COVID-19 induced syndrome of inappropriate antidiuretic hormone (SIADH) could contribute to the exacerbation of psychiatric symptoms [10]. There have been instances of COVID-19 associated catatonia linked to other pathophysiological mechanisms. One report in particular highlighted acute excited catatonia with autonomic instability associated with anti-neuronal IgG antibodies directed against neuronal targets, indicative of autoimmune encephalitis [11]. The relationship between coronaviruses and psychotic disorders is one that has been observed even prior to the COVID-19 pandemic. A case-control study published in 2009 by Severance et al. compared the rates of IgG seropositivity against certain strains of coronavirus between cases who had recently developed psychotic symptoms and non-psychiatric controls, finding that IgG seropositivity against all four strains was higher in cases [13]. 

Catatonia during the pandemic has also been noted even in the absence of COVID-19 infection. One patient developed retarded catatonia in the setting of a major depressive episode largely brought about due to concerns about the economic effects of the pandemic [14]. Other cases have been noted in patients with no prior psychiatric history presenting with sudden-onset delusional or brief psychotic disorders, likely borne from increased anxiety as in the aforementioned case and/or depression related to increased social isolation and other psychosocial stressors [15]. 

The mechanism by which catatonia occurs and more specifically how COVID-19 contributes to its development remains unclear. Given the neurotropic nature of coronaviruses, it is possible that there is a direct neurotoxic effect [16]. Glial cells and neurons have been shown to express angiotensin-converting enzyme 2 (ACE2) receptors, allowing coronaviruses to bind to them after accessing the brain either through hematologic spread across the blood-brain barrier or direct spread through the cribriform plate [17]. Furthermore, the role of the acute phase response cannot be underplayed; it should be noted that there is an upregulation of inflammatory cytokines in depression, which also occasionally presents with catatonia [15]. Damage to neurons secondary to hypoxia should also be considered; while catatonia is a rare occurrence after cerebral hypoxia, delayed neurological syndromes occurring 40 days after hypoxia have been documented [18]. Damage to neurons through the aforementioned mechanisms may explain the development of catatonia through alteration of neurotransmitter pathways. It appears to be the case that GABAergic transmission is hypofunctional in catatonia, leading to glutamatergic hyperactivity, and this explains the therapeutic effects of a GABAergic drug like lorazepam as well as NMDA antagonists like amantadine. But the overall picture is more complicated as dopaminergic and even serotonergic pathways have been implicated [6]. 

Our patient displayed at least three of the signs needed to diagnose catatonia - namely, agitation, rigidity, and echolalia - and had a therapeutic response to lorazepam, confirming our suspicions of excited catatonia. She had begun behaving bizarrely shortly after she and her husband had fallen sick with a sudden worsening of her symptoms two months after she had recovered from her “cold.” This, combined with her seropositivity for IgG against COVID-19, raised concerns for a COVID-19-induced brief psychotic disorder with catatonic features. The sudden weight loss gave rise to concerns for malignancy, but cancer screenings were up to date and the patient had regained most of her weight on follow-up. Given the patient's resolution of symptoms on follow-up, late-onset schizophrenia was considered less likely. Delirium is also less likely given the duration of symptoms, intact orientation, and positive response to benzodiazepines. In fact, benzodiazepines are not generally indicated for non-alcohol-withdrawal-related delirium and can actually worsen delirium [19]

Some cases, as noted above, have been described of patients developing brief psychotic disorders, possibly due to social and environmental stressors related to the pandemic. Given the patient's husband’s observation that they had been increasingly isolated socially, it is possible that the social isolation could have independently contributed to her psychotic episode, but considering the timing of her symptoms beginning shortly after her COVID-19 infection, it is more likely that the infection itself was a triggering or causal factor. As discussed before, the impact of inflammation and autoimmune processes within the brain cannot be understated, especially within the context of symptoms persisting or even manifesting past resolution of infection. Recent reports have suggested that autoreactive antibodies have been found in cerebrospinal fluid targeting various neural epitopes, which may provide an explanation for persistent or delayed neurological sequelae of COVID-19 [20]. 

## Conclusions

While there have been cases documented in the literature of patients presenting with catatonia during COVID-19 infection, we present the first case, as far as we know, of a patient presenting with delayed-onset catatonia after COVID-19 infection, adding to the list of delayed neurological sequelae associated with COVID-19. Given the neuropsychiatric complications that are being noted in association with COVID-19 and even prior coronavirus strains, clinicians should, after ruling out more plausible stressors, suspect possible coronavirus involvement in sudden-onset psychotic disorders, especially in patients who do not fit the demographic of new-onset schizophrenia-spectrum diagnoses. Prompt recognition and treatment with benzodiazepines can be especially important in more dangerous and life-threatening subtypes such as malignant catatonia. Finally, more research needs to be conducted into elucidating the pathophysiology behind COVID-19 and other coronaviruses altering neuronal function and neurotransmitter pathways. 
